# Hotspots and trends in health-oriented physical literacy research: a visual analysis based on the WOS database

**DOI:** 10.1186/s12889-024-18951-7

**Published:** 2024-06-03

**Authors:** Xinyuan Fang, Zhen Zhang

**Affiliations:** College of Physical Education and Health, East China Normal University, Shanghai, 200000 China

**Keywords:** Physical literacy, Health, Life quality, Physical inactivity

## Abstract

**Background:**

The World Health Organization has proposed that physical activity is a meaningful way to improve the quality of human life and reduce the probability of chronic non-communicable diseases and that humans should change their mindset from the actual effectiveness of physical activity in promoting health to the new view that “physical activity makes life more meaningful.” The introduction and development of physical literacy reveal the critical role of physical activity in improving human health and the importance of human initiative in physical activity for healthy development. Therefore, the objectives of this paper are (1) to conduct a bibliometric analysis of the literature on physical literacy, assessing the scope, frequency, and geographical distribution of research publications from various countries and institutions from 2015 to 2023; (2) to visualize keywords in articles on the topic of Physical literacy to analyze whether there is a link between physical literacy and health, and (3) based on the results of the visual analysis, we propose that proper health is built on the sense of physical literacy and further construct the circular path of physical literacy, physical activity, and physical health improvement.

**Methods:**

Using VOSviewer software v.1.6.18, this study searched the core collection of the Web of Science database from 2015 to April 15, 2023, using “physical literacy” as a keyword to explore the current international research on physical literacy.

**Results:**

A total of 3,446 articles were included, and a correlation map was derived based on the co-occurrence frequency of keywords, which showed that physical literacy was highly correlated with six concepts: health literacy, physical activity, health, children, adolescents, and prevention.

**Conclusion:**

Based on the analysis of literature visualization techniques, there is a high correlation between physical literacy and health, and international physical literacy research is in a trend of multi-point amplification, with research hotspots gradually shifting from the field of sports to the field of health and closely related to the field of health, indicating that physical literacy aims to promote the achievement of individual health by driving humans to increase physical activity.

## Introduction

In recent years, the rapid advancement of science and technology has led to the proliferation of automation, networking, and intelligent technologies in all major areas of human life and production [1]. These technologies gradually replace human physical activities, making a sedentary lifestyle the norm for many professionals and students [2]. This lack of physical activity has given rise to a global epidemic of physical inactivity disorder. According to the Global Physical Activity Report 2022 by the World Health Organization, 80% of adolescents and 27.5% of adults worldwide do not meet the recommended level of physical activity [3]. Modern society has trapped humans in a “fast-paced,” “stressful,” and “inactive” lifestyle, leading to frequent physical and mental health issues and a surge in “physical inactivity disorder “ [4]. This significantly reduces the quality of life and is associated with a range of chronic non-communicable diseases and premature death [5]. If the current global situation persists, nearly 500 million people are projected to suffer from heart disease, obesity, diabetes, and other chronic diseases due to physical inactivity between 2020 and 2030 [6].

In light of this, the report urges countries to incorporate physical activity into health-related policies as a strategic approach to improving health and tackling chronic diseases. Physical literacy, an emerging field of physical activity research, was reinterpreted by British scholar Whitehead in 1993 from a philosophical basis of phenomenology, existentialism, and embodied cognitive theory. This sparked a global surge in physical literacy research. The rapid spread of “physical literacy” in today’s world is primarily due to its novel approach to addressing the current health crisis that human society is struggling to cope with [7].

### A note of physical literacy

The term “Physical literacy” first appeared in an article published in the *American Journal of Health and Physical Education* in 1938 [8]. However, it received little attention throughout the 20th century. In the present age of technology, where machines increasingly replace manual labor and simplify lifestyles, there is a crucial need to contemplate the significance of physical activity. If physical activity is solely considered as a means to promote physical health, there is a risk of overlooking its intrinsic value, particularly for the younger generation, which generally enjoys good physical and mental health [9]. Consequently, the pressing question of how to integrate physical activity as a “necessity” in daily life and a regular part of routines arises in the 21st century, an era marked by technological transformation [10]. In response to this question, “physical literacy” has gained prominence. Although the definition of physical literacy varies internationally, it generally aligns with the summary provided by the International Physical Literacy Association (IPLA) in 2017: Physical literacy empowers individuals with the motivation, confidence, physical competence, knowledge, and understanding to value and engage in physical activity as a lifelong habit [11]. This perspective attempts to shift from viewing physical activity as a means to an end to a subjective experience that individuals actively integrate into their pursuit of life values, potential development, and quality of life enhancement. By doing so, it aims to counteract the current decline in physical activity among humans and breathe new life into human health.

### Physical literacy: a holistic approach to health and well-being

Physical literacy has received widespread academic attention because of its unique value in human physical and mental development. As an instrumental concept, it has led to a change in the thinking of physical education teachers, sports coaches, doctors, and other people concerned about physical and mental health development and influenced the formulation of related policies [12]. However, due to its complex philosophical basis and the holistic nature of mind-body unity, how physical literacy is promoted through physical activity depends mainly on the current interpretation and application of its concept in the educational community [13]. Physical literacy challenges the conventional dualism that separates the body from the mind. Instead, it espouses a monistic perspective, asserting the unity of mind and body [14]. This viewpoint posits that our physical experiences are intrinsically interwoven with our cognitive and emotional states. This monistic stance integral to physical literacy contests the traditional dualistic approach prevalent in education and health, which often segregates physical and mental development into disparate domains, completely marginalizing our body [15]. By championing the interconnection and mutual dependence of physical and mental facets of human experience, physical literacy fosters a more comprehensive, holistic approach to human development and well-being [16]. This philosophical underpinning of physical literacy has far-reaching implications for understanding, promoting, and engaging in physical activity [17]. It implies that physical activity is not merely a means for physical health enhancement but a critical element of overall personal development, cognitive function, emotional well-being, and social engagement. Therefore, the interpretation and application of physical literacy in education and health should embody this monistic philosophy [18]. It necessitates an educational and healthy approach that equally prioritizes physical and mental development, integrating physical activity into the broader context of personal growth and holistic well-being.

The definition of health has been the subject of extensive scholarly focus. According to the World Health Organization, health encompasses a four-dimensional state comprising physical, mental, social adaptability, and moral perfection. Stainton Rogers proposed that health is synonymous with “right living,” spiritual well-being, and divine care [19]. On the other hand, Schad defined health as an individual’s lifelong capacity to sustain a perfect life [20]. Interestingly, older Chinese Americans perceive health as a “balance of yin and yang” [21]. From the perspective of the British Greeks, health is an individual’s ability to work, perform household chores, and fulfill social obligations [22]. Mckague conceptualized health as a person’s capacity to meet expectations [23]. In recent years, an emerging viewpoint links health and well-being, suggesting that health is a state where physically fit individuals can effectively manage events and avoid undesirable states [24]. This perspective is rooted in a vision of human flourishing. In synthesizing these varied definitions, we contend that the essence of health aligns with the World Health Organization’s proposal of a four-dimensional state of completeness.

The realization of individual health requires attention and practice in schools, and leading students to understand the value of physical activity for physical and mental health is the basis for improving physical literacy and is the key to promoting individuals to develop a lifelong philosophy of physical education [25]. Schools should lead students to experience the enhancement of physical activity for individual well-being and to gain knowledge and understanding of the principles of holistic health to develop a clear position on the value of physical activity in enhancing overall health and well-being [26]. There are many aspects to maintaining overall health through physical literacy, including respecting the physical nature of the human condition, monitoring physical and mental well-being, building a balanced life comprised of various interests and activities, and finding a balance between new challenges and existing habits. Starting with a well-founded “standpoint” in the field of movement to understand opportunities can bring significant value in assessing personal well-being and making life choices [27].

Within the educational community, much of the literature addresses the dangers of physical inactivity disorders from the perspective of physical inactivity, with the most frequent being the serious academic consequences of physical inactivity [28]. The primary reason for the current social concern about physical health and people’s well-being is that due to the lack of physical activity, related physical activity deficiency disorder diseases are gradually appearing in more and more people, creating enormous financial pressure on the healthcare security systems of various countries [29]. This expense is borne to a large extent by people’s taxes, leading people into a physical activity deficiency disorder-access to health care-financial stress-taxation-decreased well-being circular path [30].It is evident that technology-driven society has led to an increasingly severe lack of human physical activity, and the lack of physical activity has a significant impact on human culture, healthcare, finance, education, and other fields [31], which seriously affects the realization of the vision of human prosperity. Less study based on bibliometric analysis has summarized the research progress of physical literacy from recent decades.

Utilizing bibliometric analysis, a methodology grounded in mathematical and statistical techniques, this study undertook a systematic review of articles pertaining to physical literacy over a span of time. The analysis presents comprehensive data on the countries or institutions producing these articles, co-authorship patterns, and the frequency of keywords crucial for deciphering the hotspots and trends in physical literacy research [32]. Collaboration among countries, authors, and institutions was scrutinized through tables and graphs to better discern the leading contributors to physical literacy research. This approach aids in comprehending the areas that have made significant progress and contributions. Furthermore, an examination of keywords related to physical literacy was conducted. Finally, publication trends in literature associated with physical literacy were explored using line charts. Through this study, our objective is to offer a more comprehensive and intuitive understanding of the shifts in developmental trends and popular research directions within international physical literacy research. This will serve as a valuable reference for shaping the future direction of physical literacy research.

## Methods

### Search strategy

To ensure the authority and scientific validity of the research subjects, the data source for this analysis was the Web of Science core collection (including SCIE/SSCI/A&HCI and ESCI, etc.) as the data source, which derived from Clarivate Analytics, contains more than 12,000 international academic journals and is recognized as a comprehensive and authoritative database [33].The data search strategy was as follows: (1) Subject="Physical literacy”; (2) Document type= (review or article); (3) Language="English”; (4) Search date = From January 1, 2015 to April 15, 2023. All data were obtained on April 15, 2023. A thematic search was used to balance accuracy and completeness, and a total of 3,439 documents were extracted. VOSviewer software v.1.6.18. was used to analyze the sample literature in order to obtain the evolutionary relationship between hotspots and trends in physical literacy research. For better use in VOSviewer analysis, this study exported the documents as Plain Text File with Record Content of Authors, Title, Source, Times Cited Count, Accession Number, and Abstract. The processing parameters were set as follows: annual interval 2015–2023, time slice of 1 year, and thematic sources as full citations and citation references.

### About text preprocessing

VOSviewer software emerges as a robust tool for bibliometric analysis, boasting unique features that negate the necessity for extensive data preprocessing. Its strength lies in the seamless integration of the VOS mapping technique and an advanced viewer into a single, user-friendly computer program. With a distinct emphasis on graphical representation, VOSviewer offers an intuitive display for large bibliometric maps, as demonstrated in constructing a co-citation map involving 5,000 major scientific journals [34, 35]. Notably, its efficiency in capturing current trends in science and technology surpasses traditional methods, evidenced by the swift analysis of articles, papers, and patents. Nees Jan van Eck and Ludo Waltman extensively explore the program’s functionality and technical intricacies in their respective papers [36].

Furthermore, VOSviewer’s inclusion of text mining functionality empowers users to analyze extensive textual data efficiently. Despite encountering some missing values and minimal anomalies, a deliberate decision was made to forgo data preprocessing, aiming to preserve the raw state of the data for a more authentic representation of real-world scenarios. Acknowledging the potential impact of unprocessed data on experiment results, this approach is chosen to provide a more genuine perspective on physical literacy in our study. This decision aligns with VOSviewer’s capabilities, making it a versatile and comprehensive tool for researchers and analysts in bibliometrics.

### Date analysis

In analyzing the data, we evaluated the following aspects: (1) Country and institution of publication, analyzing the current status of international research on the topic by country and institution of publication; (2) Years of publication, highlighting the focus of the literature at different time points; (3) Concentration of keywords in the articles, highlighting the most frequently used keywords in the article collection; (4) Authors with the most published articles and the nationality of the authors; (5) WOS categories, analyzing the yearly trend of the literature on the topic of “physical literacy” and the correlation coefficient. (6) WOS categories, analyzing the publishers, publishers, and journals; (7) The annual trend of publications on the theme of “physical literacy” and the correlation coefficient analysis.

## Results

### Country & institution analysis

**Fig. 1:**
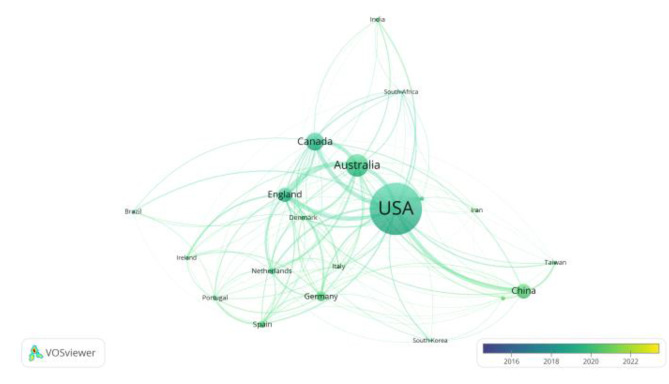
Country co-existence time analysis map

**Fig. 2:**
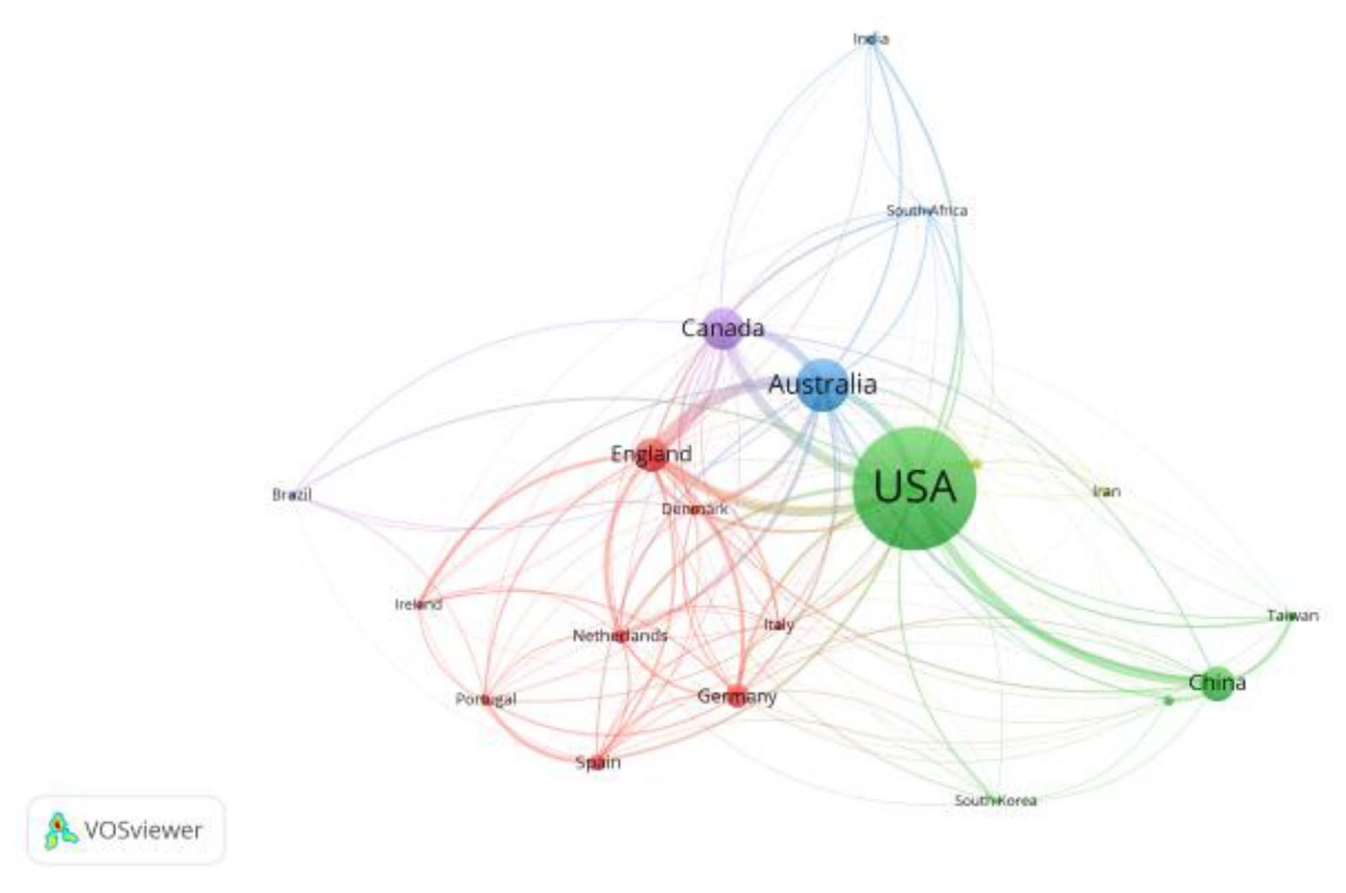
Country co-existence map

The country was selected as the node type and ran to obtain a network map of the current international geographic contributions to physical literacy-related research. In the knowledge map, the size of the circle represents the citation history of the topic, and the larger the circle is, the higher the frequency of the topic’s appearance. First, regarding country analysis in Figs. 1 and 2, the highest number of articles was 1,070 in the United States, 439 in Australia, 343 in Canada, and 270 in the United Kingdom. In terms of article relevance, the highest U.S., Canada, UK. In summary of the data the United States and the United Kingdom have more significant research on physical literacy and stronger correlations between them, indicating that the two countries have more theoretical and practical achievements in physical literacy research. From the analysis of its theoretical level, the theory of physical literacy was laid by the British scholar Whitehead from the philosophical level. The US research-related theories are primarily based on Whitehead’s theory of physical literacy. At the same time, Canada and Australia have developed many physical literacy assessment tools related to CAPL and PFL, which undoubtedly triggered the research enthusiasm of scholars in related fields.

**Fig. 3:**
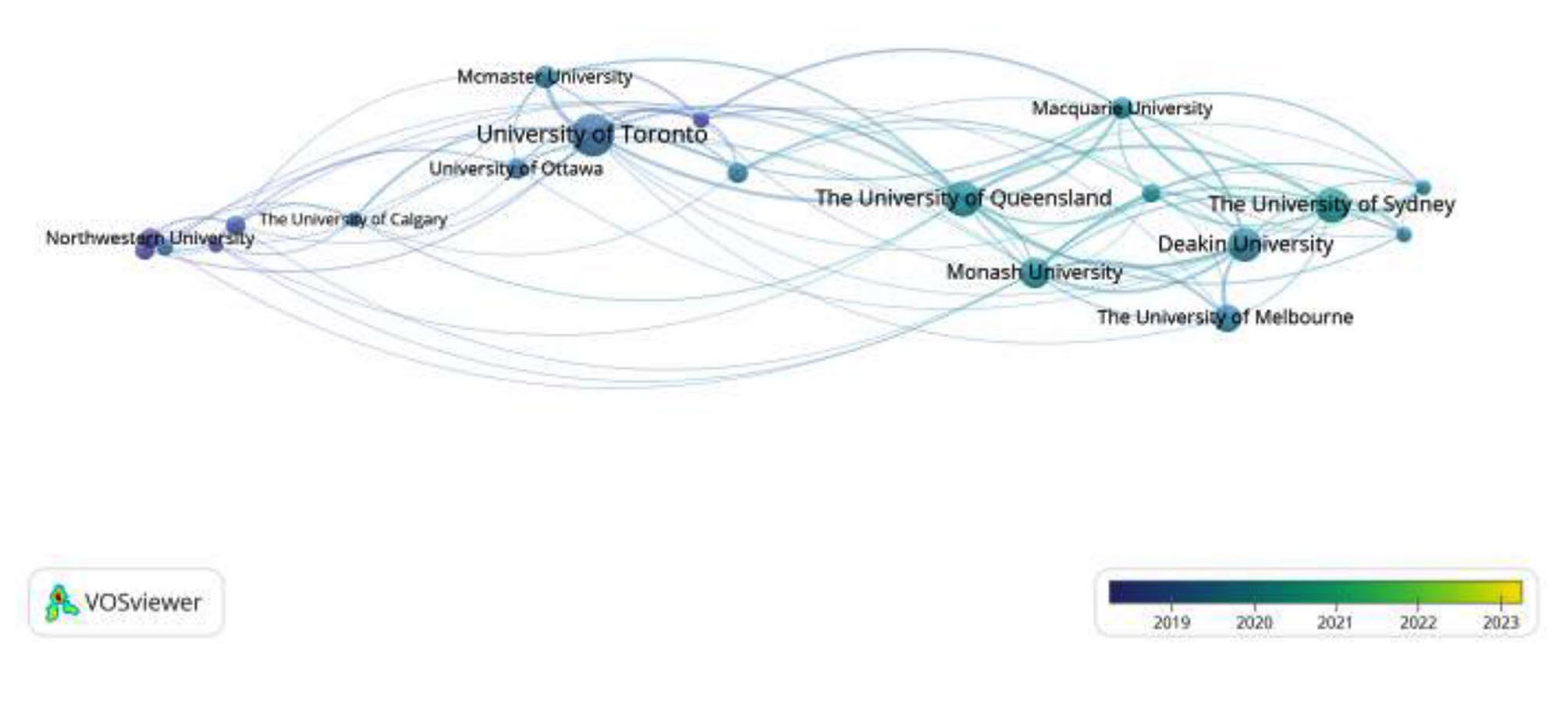
Issuing institutions time map

**Fig. 4:**
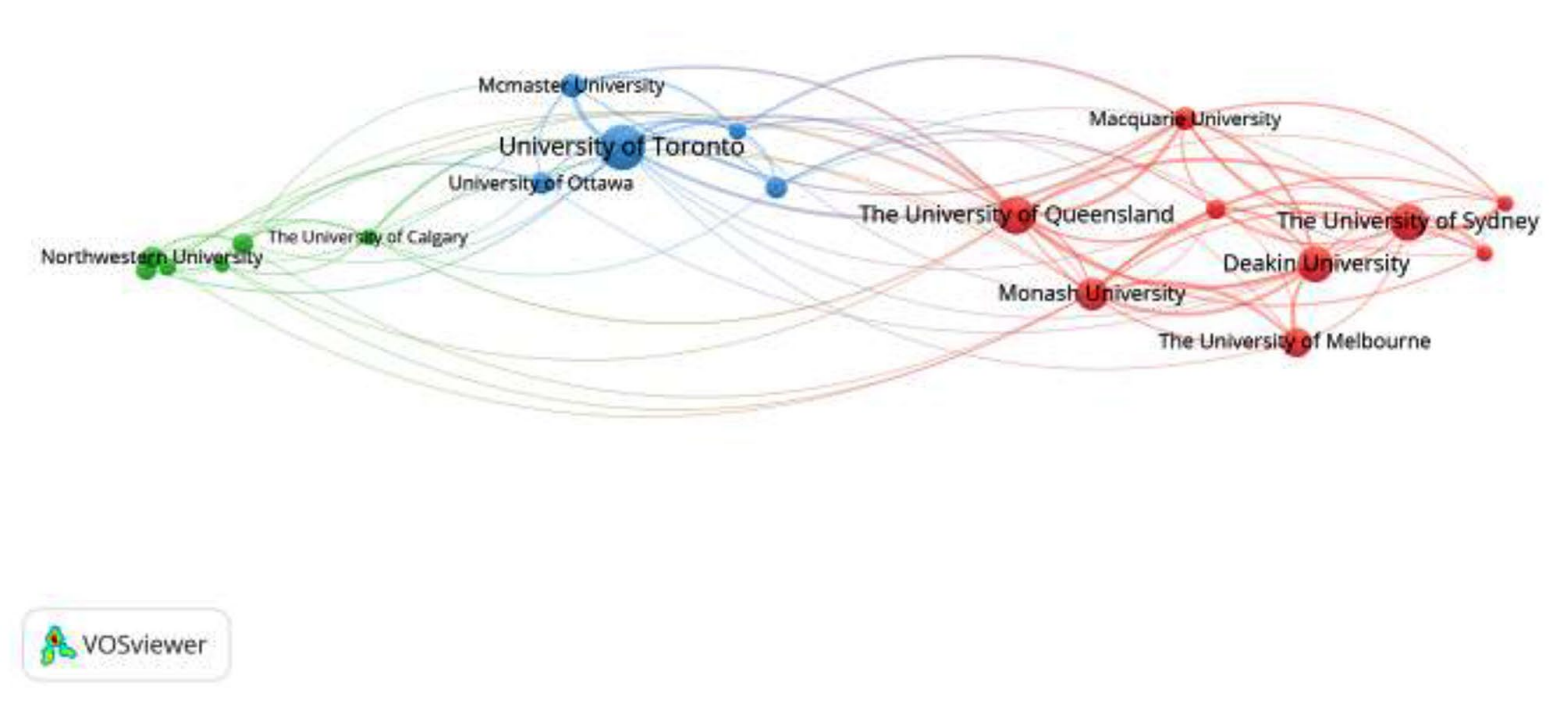
Issuing institutions co-current map

In terms of the institutions that publish articles in Figs. 3 and 4, higher education institutions are the main force of physical literacy-related research, concentrated in the United States, Canada, Australia, and other developed countries’ top universities, mainly including the University of Sydney, Deakin University, Stanford University, Columbia University, Newcastle University, Flinders University, University of California, University of Ottawa and so on. These universities have a medical and sports science-based. The authors of these articles are primarily students or faculty members of these two fields of study.

### Keyword co-occurrence analysis

**Fig. 5:**
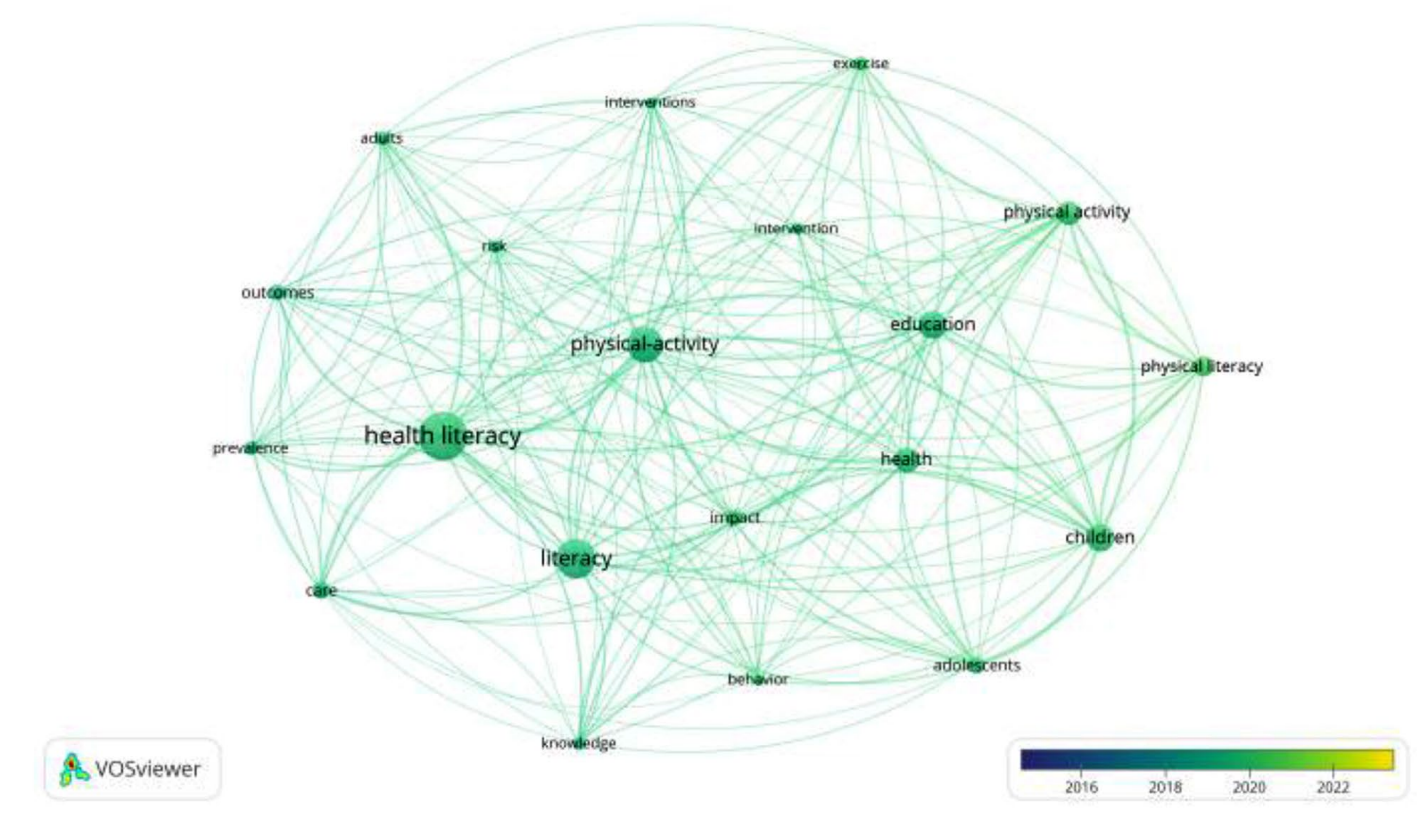
Keyword co-occurrence map

**Table 1:** Frequency of keyword co-occurrence in the literature

Key words	Co-occurrence frequency	Frequency probability
Health literacy	608	0.18
Physical-activity	442	0.13
Children	341	0.10
Physical activity	298	0.09
Literacy	496	0.14
Education	343	0.10
Health	315	0.09
Physical literacy	258	0.08
Adolescents	210	0.06
Impact	203	0.06
Exercise	172	0.05
Care	217	0.06
Outcomes	199	0.06
Adults	179	0.05
Interventions	144	0.04
Knowledge	165	0.05
Behavior	144	0.04
Intervention	151	0.04
Risk	161	0.05
Prevalence	178	0.05

Keywords are usually the core content and methodological refinement of a paper, and the frequency of keywords in a field can visually reflect the distribution of research hotspot directions centered on that topic. The keyword network map of current physical literacy-related research was run by selecting the node types as keywords in Fig. 5; Table 1. It can be seen that among the current literature on “physical literacy”, international research mainly focuses on adults, adolescents, and children, including health literacy, health, physical activity, exercise, prevention, behavioral habits, literacy, and other aspects. The time zone analysis chart more clearly shows the evolutionary path of the hotspots of physical literacy research. Although the formal definition of physical literacy is concise, its theoretical research has developed very fast, from the early focus on children and adolescents’ physical activity to the gradual development of individual physical literacy through education and the proliferation of the curriculum, policies, measurement tools, skills and knowledge, after which physical literacy is often associated with issues such as health levels, motivation, public health, physical activity deficit disorder, and human flourishing. Physical literacy and human health have recently become an emerging trend in international scholarship. In terms of changes in research hotspots, over time, research on physical literacy has seen a shift from a focus on individual motor skills to a focus on human health, and overall, physical literacy has become increasingly relevant to physical health as an essential literacy for human health.

### Main authors and number of citations

**Fig. 6:**
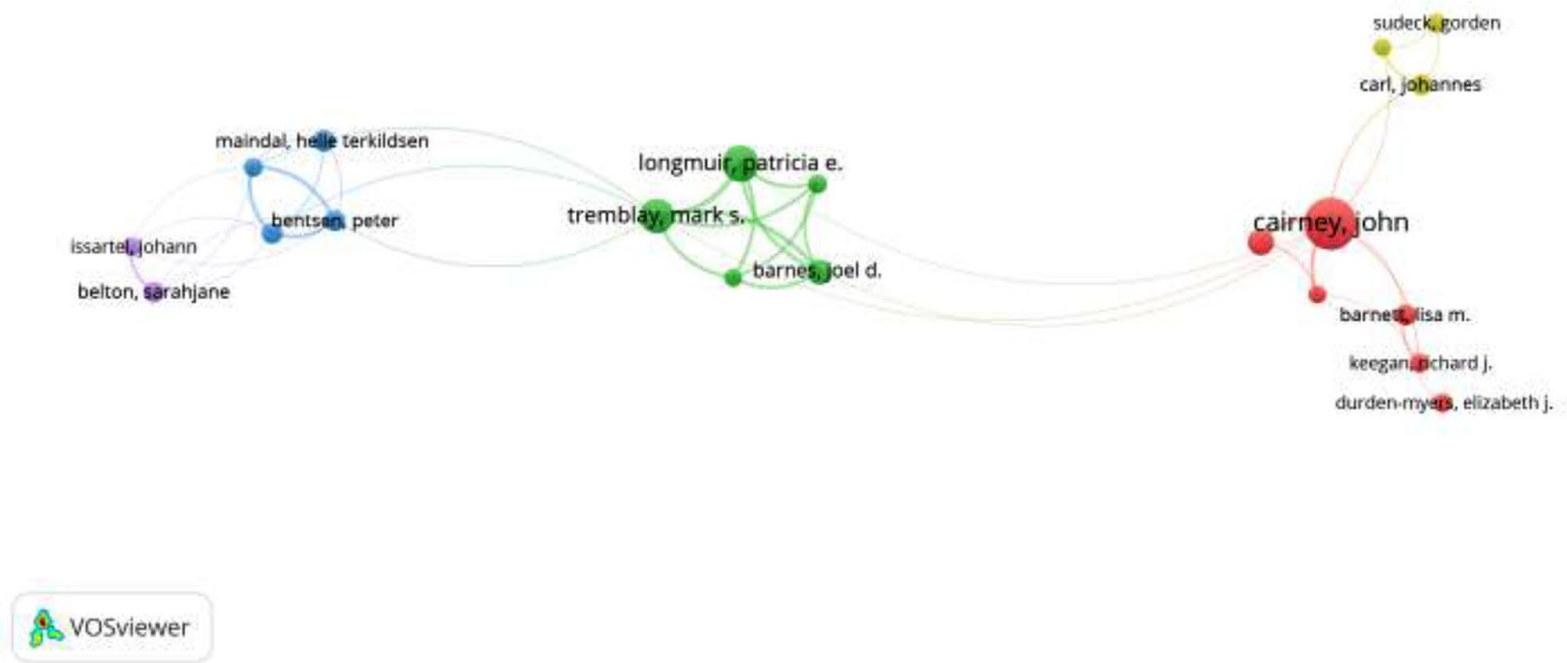
The first author of the article co-presents the figure

Figure 6; Table 2 show the authors who have published more papers in “Physical Literacy”: Tremblay, Mark S. and Longmuir, Patricia E. from Canada with 23 and 24 papers, respectively, and Cairney, John from Australia with 36 papers. From the figure and table, we can learn the number of articles published by the first author of the paper and the number of citations to the article.

**Table 2:** Authors with the highest number of publications and citations

Authors	Country	Doc.	Cit.
Cairney, John	Australia	36	664
Longmuir, Patricia E.	Canada	24	622
Tremblay, Mark S.	Canada	23	723
Kriellaars, Dean	Canada	17	413
Barnes, joel D	The USA	16	444
Wolf Michael S.	The USA	15	174

Doc. (Documents); Cit (Citations)

### WOS categories

In the WOS core collection, all literature is divided into 191 categories, of which health and wellness is the category with the most significant number of articles (750), followed by education and teaching research (571), sports science (302), and health care science services (246). Table 3 shows, in addition to the categories with the highest number of articles, the journals and publishers with the highest number of papers published under the title “physical literacy.” The journal with the most articles was the International Journal of Environmental Research and Public Health, with 147 articles. In contrast, the publisher with the highest number of articles was Springer Nature, with 498 articles.

**Table 3:** Authors with the highest number of publications and citations

WOS Categories	Doc.	Prolific Journals	Doc.	Prolific Publishers	Doc.
**Public Environmental Occupational Health**	750	**International Journal of Environmental Research and Public Health**	147	**Springer Nature**	498
**Education Educational Research**	571	**BMC Public Health**	97	**Taylor & Francis**	379
**Sport Sciences**	303	**BMJ Open**	52	**Elsevier**	313
**Health Care Sciences Services**	246	**Journal of Medical Internet Research**	41	**MDPI**	259
**Environmental Sciences**	197	**Frontiers in Psychology**	40	**Sage**	251

Doc. (Number of documents)

### Annual publication trends

**Fig. 7:**
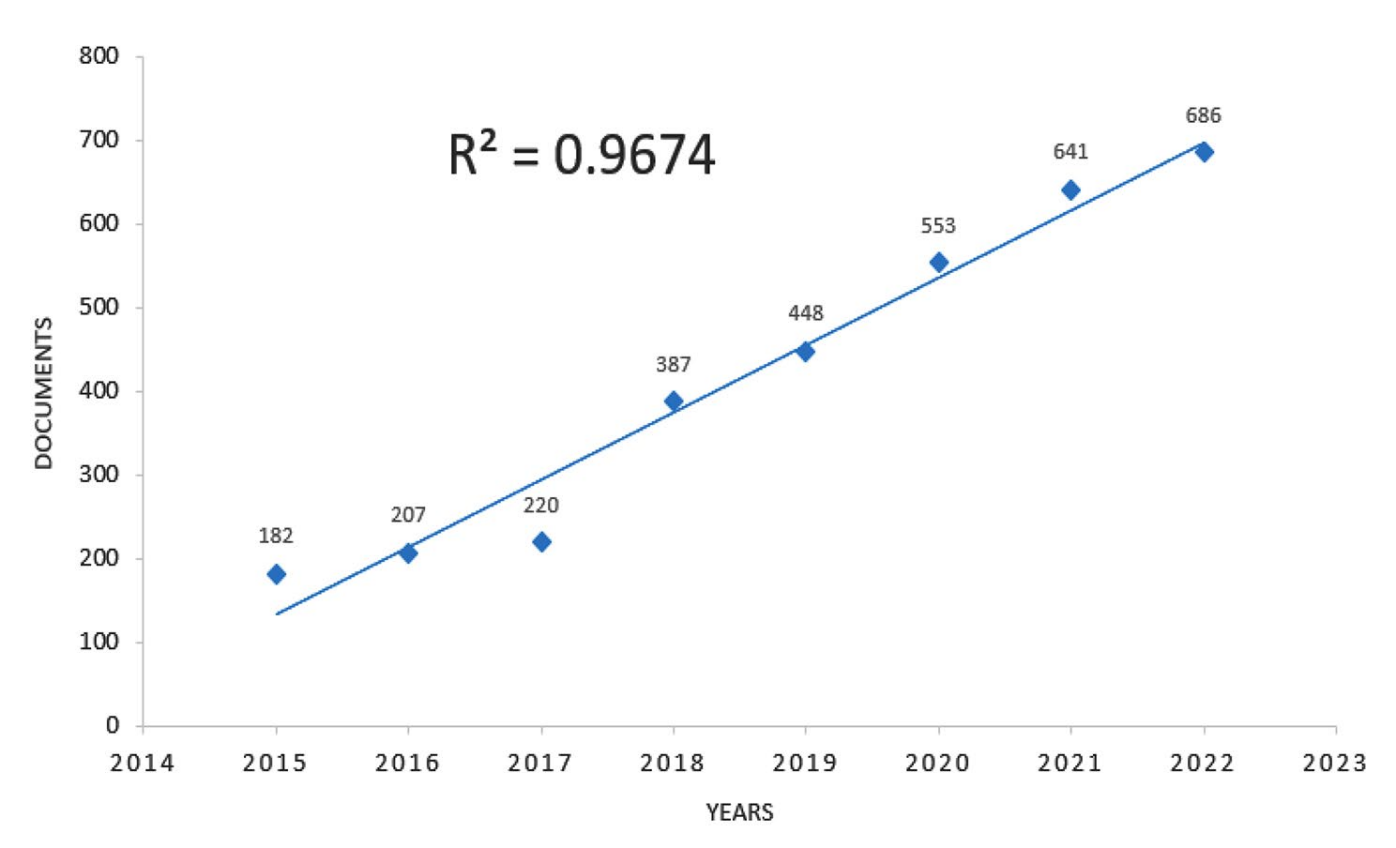
Trends in publications

Through the WOS core database, we found 3,446 articles published from 2015 to April 15, 2023, with 2022 being the most published year, with 686 articles published on “physical literacy.” Since 2015, the literature on “physical literacy” has shown an exponential increase with a fit rate of R²=96.74% in Fig. 7.

## Discussion

This study aimed to analyze current international trends in physical literacy and research hotspots to provide a comprehensive and practical review to the scientific community in a visual manner. A bibliometric study of articles on physical literacy revealed that research on physical literacy and health has evolved rapidly from establishment to emergence. The various definitions of health provide a broad context, yet the concept of physical literacy, a critical aspect of overall health, still needs to be more adequately explored. Although Cairney constructed a circular argument framework based on health, physical literacy, and physical activity, physical literacy has yet to receive much attention [37]. This may be because the term “health literacy” has been commonly used in the academic community to describe the ability of individuals to maintain their physical health actively. Physical literacy is believed to be considered health literacy in the medical field [38]. Therefore, this study analyzes the relationship between “Physical literacy” and “Health literacy”. Based on these analyses, we investigate the concept of physical literacy as a meaningful concept based on physical activity and further argue for the circular enhancement pathway of health, physical literacy, and physical activity proposed by Cairney et al.

### General information study

The upward trajectory of scholarly output on physical literacy since 2015, peaking in 2022, underscores the burgeoning interest within the academic community. Predominantly, developed nations such as the United States, Canada, Australia, and the United Kingdom have been at the forefront of this discourse, as depicted in Fig. 1. The United States, in particular, exhibits a robust interconnection with international research efforts, signifying its pivotal role in advancing physical literacy scholarship. Regarding institutional contributions, a concentration of seminal work emanates from developed countries, with prestigious entities like the University of Sydney, Deakin University, Stanford University, and Columbia University leading the charge. This confluence of geographic and institutional provenance indicates that developed nations have established a significant lead in physical literacy research, outpacing their counterparts in the developing world, where research in this area is emerging and needs augmentation.

The disparity in research output can largely be attributed to the superior financial and infrastructural provisions available in developed countries, which foster environments rich in high-caliber research institutions, cutting-edge laboratory facilities, and comprehensive funding opportunities for physical activity research. Conversely, developing nations often grapple with resource allocation challenges, necessitating prioritization of immediate societal needs such as infrastructure over research endeavors. Moreover, the academic milieu in developed countries is inherently conducive to generating high-quality research, bolstered by a tradition of frequent academic discourse and international collaborations that enhance both the caliber and the innovative potential of scholarly work on physical literacy.

An analysis of the 3,439 articles published reveals that the top five journals account for 337 publications, representing approximately 11% of the total volume. John Cairney stands out as the most prolific author in the field, extensively exploring the interconnections between physical literacy, health, and physical activity, often employing a cyclical argumentative structure to encapsulate these interdependencies. The International Journal of Physical Literacy and Physical Activity is a frequent publisher in this domain. Meanwhile, the International Journal of Environmental Research and Public Health boasts the highest publication frequency (0.4%), reflecting a health-centric journal’s predilection for physical literacy topics, corroborated by the keyword analysis in Fig. 5; Table 1. The analysis confirms a robust association between the literature on physical literacy and health-related themes, providing insights into the evolving trends and focal areas within the field. In the subsequent sections, the focus will be on the relationship between physical literacy and health.

### Physical literacy and physical activity

The development of physical literacy allows participation in lifelong physical activity through improved motor skills and cognitive-emotional abilities [39]. From a biological perspective, life is how living things exist in the world, and as social beings, individual lives cannot meet the needs of social life. Therefore, to view life from the perspective of physical literacy is to perceive the world in terms of physical activity, to experience the meaning of life, and to relish it. Physical activity is ubiquitous in life; life is the basis of life, and the integrity and health of life depend on the subjective activities of human beings. Physical activity is the basis for human beings to achieve freedom and comprehensive development of life [40].

At present, the educational concept that physical literacy can effectively promote the healthy growth of youth and that physical activity can make life more meaningful has become a global consensus [41]. However, physical activity is not synonymous with childhood nor an exclusive term in education, but it is all those human beings do in their daily lives. Physical literacy plays a significant role in human beings’ lifelong physical participation and healthy development [42]. As actual human beings, we need to see ourselves as an existential whole, which is, in essence, how we interact with the world, that is, when the more physically active we are, the better we perceive the world [43], physical literacy provides a rational explanation for physical activity, and technological developments in contemporary society have increased the demand for physical activity, and a study has shown that An effective way to improve the level of physical literacy is to reduce the amount of sedentary time [44], this is because, while physical activity was once used to meet the needs of material life, physical activity nowadays is more about improving the quality of human life in terms of spiritual life, and although the human civilization system constructed by modern society has cultivated the inner qualities of rationality and solidarity, it has also to some extent suppressed the instinct of human beings as animals, which is the ability to be strong through physical skills to demonstrate muscular physique, proficiency, and thus spiritual satisfaction [45]. Physical literacy, as a lifelong concept, is proposed not only for the healthy growth of youth but also for the vision of prosperity of all human beings; its essence is the development of the individual and the realization of personal potential through physical activity to promote social development. Therefore, this section analyzes how physical literacy and physical activity are developed based on the elaboration of the role of physical literacy in the whole life cycle [20].

**Table 4:** Constructional map of physical literacy and physical activity throughout the life cycle

Physical Literacy and Physical Activity(From Cradle to Grave)						
**Physical literacy is the fundamental goal of life care education**					**Social Stage**	**Senile stage**
**Preschool stage**	**Primary school stage**		**Secondary school stage**	**University** **Stage**		
Encourage and support physical activity	Cultivate the formation of **basic sports patterns** and understand **physical knowledge**		Continuously improve **physical activity abilities** in situational learning and socializing	Regularly participate in **physical activities** to **maintain** physical health and improve **physical literacy** as an integral part of extracurricular life	Establishing physical literacy, actively integrating physical activities into **daily life**, treating them as a **necessity**, and **reducing sedentary behaviors** to promote **overall health**	**Reducing** physical activity **intensity**, developing suitable activity styles based on current **physical condition**, deepen understanding of elderly physical health changes, and promoting **positive lifestyles**
**Individuals who affect the acquisition and maintenance of physical literacy**						
Parents and other important members of the family	Teachers, parents, families, peers, coaches, clubs, and leisure facility service personnel			Friends, family, colleagues, professionals in related fields (medicine, sports)		
**Promoting, establishing, and maintaining a system, setting, and environment for physical literacy**						
Family, local environment, day care institutions, preschool activity clubs		School-based activities, sports clubs, families, community resources, local facilities		Quality and quantity of local and **national sports facilities** and **personnel**, government **policies and priorities**, medical environment, media environment		

As can be seen from Table 4, physical literacy, as a concept covering the whole life cycle, has different training modes and influencing factors at different ages. The training process of physical literacy is not an overnight process but a continuous process [46]. Based on monistic physical literacy, the concept of well-being from the perspective of the whole person is upheld. Exercise mode, mental skills for self-management, and basic health and nutrition knowledge are taken as essential components to achieve physical and mental health [47]. When carrying out physical activities, every movement must go through the procedure of “perceiving the world-processing information. Therefore, developing physical literacy is realized through constant autonomous human activity, a cognitive process in which we perceive the world through our bodies and judge it through our minds [48].

### Physical activity and physical health

Guiding humans to understand the impact of physical inactivity on physical health remains the most significant challenge for the public health community today [49]. The World Health Organization has proposed that health is a four-dimensional state of physical, mental, social adaptability, and moral perfection, and whether the definition of health needs to be redefined for better understanding and use under new social conditions, physical activity provides a suitable explanation for health [50]. In 21st-century society, human beings are gradually domesticated by technology, and society is characterized by technological and instrumental rationality. Developed countries, led by the United States, the United Kingdom, and Switzerland, have been more severely affected by technology, with the United States experiencing a 32% decline in national physical activity from 1965 to 2009 and emerging countries rapidly following in its footsteps, with China experiencing a 45% decline in national physical activity from 1991 to 2009 [6].

The Lancet states that as of 2022, global progress in promoting physical activity remains stagnant, with more than 5 million deaths yearly due to physical inactivity [51]. The neglect of physical activity has led to the trivialization and even denial of physical education that promotes physical and mental health, weakening individual subjectivity and adversely affecting physical, mental, social interactions, and spiritual pursuits. The health benefits of appropriate physical activity, including reduced risk of cardiovascular disease, diabetes, and cancer, are well established [52]. Obesity, now the most prevalent disease in the world, arose from excessive energy intake and reduced energy expenditure, corresponding to unhealthy dietary habits and chronic physical inactivity, respectively [53]. It is now established that physical activity effectively reduces the risk of obesity [54]. It has been shown that dietary habits are associated with sedentary behavior, which is defined as static physical activity with low energy expenditure in a sitting, lying, or supine position, and that chronic sedentary behavior also increases the probability of non-communicable diseases and the risk of death, with sedentary time exceeding eight hours per day increasing mortality by nearly 8% [50]. The literature shows that active physical activity is strongly correlated with physical health and that regular physical activity is essential to achieving human health and improving quality of life [55].

At the same time, recent studies have reported that at least 20 min of moderate-to-vigorous physical activity per day can reduce the prevalence of cardiovascular disease in adults by 16–40% [56, 57] and that a physically inactive an active lifestyle will increase the likelihood of chronic non-communicable diseases and increase the risk of cancer, which together raise the risk of death [58]. An American College of Sports Medicine created a physical activity guide chart for different populations based on the intensity and frequency of physical activity, aiming to promote the development of national physical activity habits and the level of national physical health through this guide [59]. The level of physical activity determined the mental health of individuals, with higher intensity of daily physical activity being associated with higher mental health scores in subjects [60]. From the literature related to physical activity and physical health, it is clear that physical activity is a determinant of physical health. If a person misses physical activity, he will be unhealthy or subhealthful [50]. This can redefine the concept of health from the perspective of physical literacy. That is, health is a complete state based on physical, mental, and social adaptability and morality based on physical activity. The overall improvement of physical literacy can lead to higher physical quality and a healthier lifestyle, thus achieving comprehensive physical and mental health development [61]. Recently, Japanese scholar INOUE Kosuke proposed a 14.9% reduction in all-cause mortality in people who performed 1–2 walks of 8000 steps or more per week and a 16.5% reduction in the risk of all-cause mortality in people who took 8000 steps or more 3–7 days per week based on 10-year-long follow-up data [62].

German scholar Lars Gabrys et al. suggested that long-term physical activity could improve cardiometabolic health, while abrupt interruptions in regular physical activity would increase the incidence of cardiometabolic disease [63]. Therefore, understanding physical activity as a determinant of health within this concept of health will be vital to developing effective interventions targeting public health [64]. Good levels of physical literacy are strongly correlated with maintaining a high frequency of physical activity, with physical literacy being a key indicator of promoting physical activity and health levels in secondary school students to help them maintain an active lifestyle [65]. A recent study by Caldwell et al. demonstrated that the determinants of physical health in adolescents are their possession of high levels of physical literacy, that these adolescents have a better quality of life, and that promoting physical health through physical activity helps them maintain a low risk of prevalence of chronic non-communicable diseases in the long run [66]. Another study showed a negative correlation between the intensity of physical activity and individual BMI. Those who are regularly physically active also have the initiative in body image management [67].

### Physical health and physical literacy

Physical literacy describes “the value and responsibility of an individual’s motivation, confidence, ability, knowledge, and understanding of lifelong physical activity” [68], which is based on physical activity and integrates physical cognition and experience. Physical literacy holds a unique significance in promoting physical health, enabling individuals with proficient physical literacy to address existing health issues through physical activity and prevent the onset of diseases. In the discourse surrounding physical literacy, it is posited that its ultimate purpose is to cultivate individuals who are both healthy and holistically developed, thereby maximizing human potential [69]. The concept of physical literacy embraces the diversity of physical activities, acknowledging that not all are explicitly aimed at health outcomes. For instance, although fundamentally functional or mechanical, occupational tasks and everyday activities are pivotal in realizing value and engaging with the world as individuals interact with their environment [70]. Value is realized in mechanistic or functional physical activities. Within the framework of physical literacy, health-oriented physical activities should not be perceived as a coercive health-centric agenda. Instead, it fosters a comprehensive perspective on physical activity, recognizing multiple dimensions and benefits, with health being just one of many. The conception of health promoted by physical literacy aligns with the pursuit of human flourishing, where even mechanical or functional work is seen as an opportunity to enhance physical capabilities, confidence, and motivation. Physical activity is not solely a pursuit of health but is a pursuit of true freedom by humans as advanced intellectual animals. Physical literacy fosters a sense of bodily freedom, enabling us to navigate the world, interact with others, and fully participate in the variegated experiences of humanity [71]. Hence, mechanistic or functional physical activities are not the focus of physical literacy. Our bodies evolved to thrive in diverse environments, and advancing human technology aims to achieve greater freedom, not diminishing physical activity [2]. Our well-being is contingent on the diverse challenges and contexts within the world, and it is through engaging in these physical activities we find meaning in life and fulfill the ultimate aim of physical literacy [72].

**Fig. 8:**
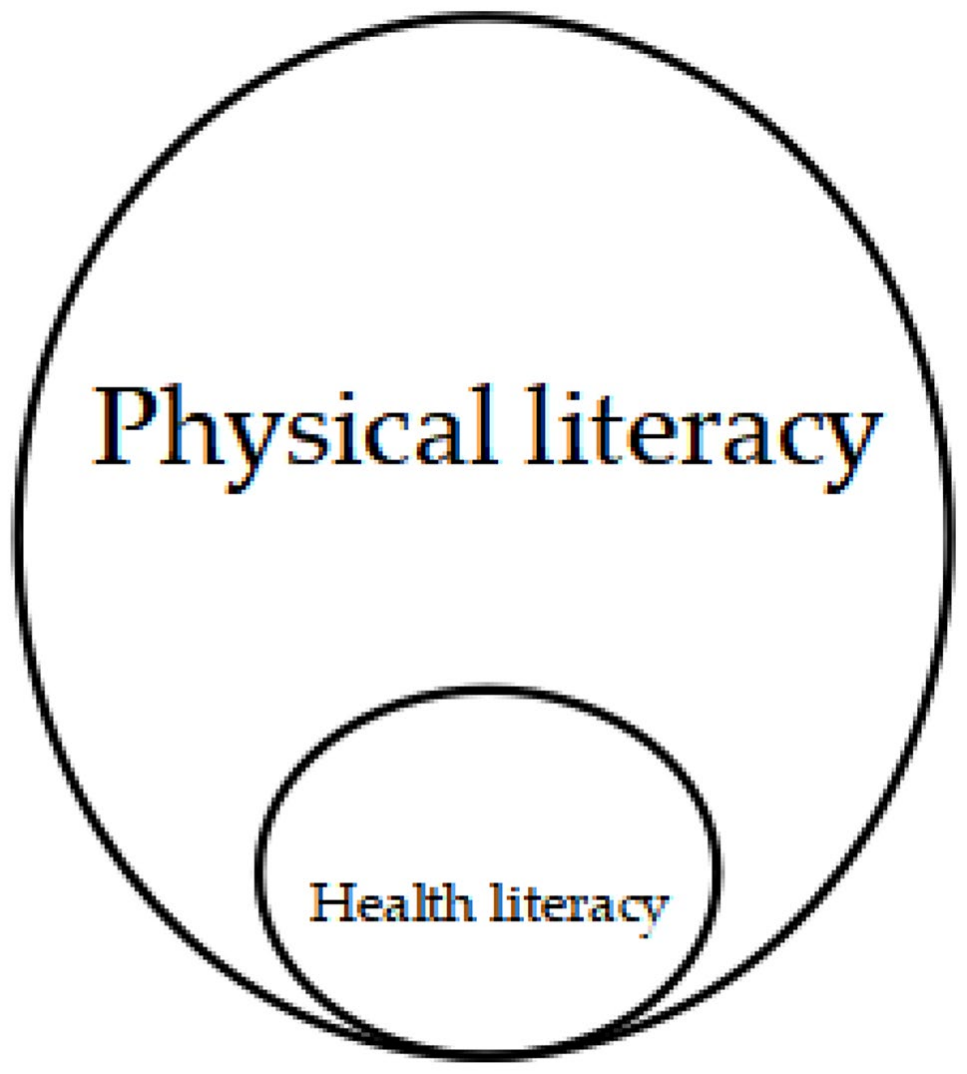
Physical literacy and health literacy

The keyword co-occurrence shows that “health literacy” still appears more frequently in the literature on “physical literacy” because health literacy has been proposed earlier. However, there is a lack of “physical activity” transformation into “fitness activity.” This is because health literacy was introduced earlier but lacks the process of transforming “physical activity” into “fitness activity,” so physical literacy is different from health literacy in Fig. 8. Chinese scholars such as Zhou Wansheng distinguished the connotation and relationship between physical literacy and health literacy from the perspective of sports ontology, pointing out that physical literacy comes from embodied human beings, which is the unity of body, mind, cognition, and behavior, and is used to describe the physical activities that human beings have to perform throughout their lives. In contrast, health literacy comes from the health education model established by human beings to promote health. Its fundamental purpose is to cultivate healthy human beings [73]. However, the ultimate purpose of physical literacy is to cultivate physically healthy and well-rounded people. Physical literacy and health literacy are interrelated, mutually influencing, and promoting each other rather than being separate individuals [74].

For many years, the Western world has been influenced by the Cartesian mind-body dichotomy, where physical activity has become less and less important, and sport is seen as a tool to achieve other purposes [75]. However, a growing body of research suggests that physical and mental development is integrated and that physical activity contributes to brain development during adolescence, where adolescents experience the world and enhance their perception of it through physical activity while also being able to develop basic motor patterns [14], which can be developed to lay the foundation for higher levels of physical activity and provide a great contribution to a full and high quality of life [76].

Therefore, the embodied and motor nature of humans is a critical pathway for our interaction with the world [77]. Developing physical literacy is fostering human initiative and stimulating individual potential, as well as developing individual self-esteem, self-confidence, and other abilities that have a positive correlation with attitudes toward physical activity [78], people with higher self-confidence and self-awareness will maintain a more positive attitude toward physical activity, physical literacy is intrinsic to life development, and there is growing evidence that the earlier the level of physical literacy is developed The more beneficial it is for physical activity in later life [14], a physical literacy development program in the United States that students should be provided with activities that are appropriate for them and allow them to feel the process of activity rather than the outcome [63], physical literacy is based on existentialism, phenomenology, and mind-body monism as theoretical foundations, and in recent years, physical literacy has been gradually viewed as an indirect factor of health, existential physical literacy, physical activity, physical health pathway, where improved physical literacy leads to more physical activity, and people with good physical literacy continuously engage in physical activity, resulting in positive physical, psychological, and social adaptations that improve individual physical health [37], based on Whitehead’s definition of physical literacy, physical literacy is true at all ages in humans [79], from infancy to old age, Hilary A.T., Caldwell et al. verified that physical literacy has a positive correlation with health through a combination of PLAY fun, PLAY self, and PLAY parent [80], and in Canada, children who met Canada’s own physical activity guidelines showed In Canada, children who met Canada’s national physical activity guidelines showed better fitness, motivation, and confidence [81], which could prove the purpose of physical literacy, which was proposed to reverse the current decline in human physical activity and to make life more meaningful and address the “existential crisis” people are currently facing [7].

## Conclusion

Our study aims to comprehensively and intuitively map the evolving landscape of international physical literacy research, offering valuable guidance for future research trajectories in this area. By visualizing the data, this study found that since 2015, the trend of exponential growth of articles on the topic of “physical literacy” and the increasing trend of research on physical literacy for health indicates that the international scientific community is increasingly interested in the research on “physical literacy and is gradually exploring the possibility of promoting human health through physical activity and changing the current trend of declining quality of life. This indicates the international scientific community’s growing interest in “physical literacy” and the progressive exploration of physical activity to promote human health and change the declining quality of life. Regarding the sources of the articles, the International Journal of Environmental Research and Public Health, BMC Public Health, BMJ Open, and Journal of Medical Internet Research, the most significant number of articles was published in the Journal of Medical Internet Research, with more than 45 articles. Regarding the keywords of the articles, these papers mainly focused on physical activity, physical literacy, and health literacy, and the number of occurrences of these three keywords was 740, 608, and 258, respectively.

It is evident that physical literacy, as an emerging concept, has become a growing and crucial topic. In recent years, physical literacy has emerged as a scene to discuss the relevance of physical activity and health, to discuss that developing physical literacy can improve various domains (physical, cognitive, mental, and emotional) in which individuals participate in physical activity so that through the improvement of individual physical literacy they can better feel the pleasure and meaningfulness, increasing human confidence in performing physical activity, and thus increasing human attitudes toward having a more active and healthy lifestyle. However, the country mapping shows that the current research on physical literacy is more in developed countries such as the United States, Australia, Canada, etc. Most countries have studied physical literacy late and have not combined physical literacy with physical health, or some countries have only used physical literacy to study specific groups (elderly, disabled), which is contrary to physical literacy as an ability that all human beings have. This is contrary to the ability of physical literacy as a human individual. Based on the above, this study further demonstrates a circular pathway between physical literacy, physical health, and physical activity based on previous research.

Despite the insights this study provides into the research hotspots and trends in physical literacy, several limitations exist. This study is confined to the WOS core collection, and while extensive, our analysis may need to be more comprehensive. Secondly, our research is limited to articles written in English as the writing language. Thirdly, the study does not take into account the quality of the publications, a factor that could have an impact on the results. Finally, although the VOSviewer is a professional bibliometric analysis software tool that provides objective analysis, there may be some subjective deviation as researchers may have different perceptions and interpretations of the same content.

## Data Availability

The datasets used and/or analyzed during the current study available from the corresponding author on reasonable request.
